# Brain, Behavior, and Cognitive Interplay in Disorders of Consciousness: A Multiple Case Study

**DOI:** 10.3389/fneur.2018.00665

**Published:** 2018-08-14

**Authors:** Charlène Aubinet, Lesley Murphy, Mohamed A. Bahri, Stephen K. Larroque, Helena Cassol, Jitka Annen, Manon Carrière, Sarah Wannez, Aurore Thibaut, Steven Laureys, Olivia Gosseries

**Affiliations:** ^1^Coma Science Group, GIGA Consciousness and Neurology Department, University and University Hospital of Liège, Liège, Belgium; ^2^Department for Neuro and Clinical Health Psychology, St George's University Hospital, London, United Kingdom; ^3^GIGA-Cyclotron Research Center in Vivo Imaging, University of Liège, Liège, Belgium

**Keywords:** (emergence from) minimally conscious state, behavior, cognitive functions, neuropsychological assessment, positron emission tomography, structural magnetic resonance imaging, neural correlates

## Abstract

Patients with prolonged disorders of consciousness (DoC) after severe brain injury may present residual behavioral and cognitive functions. Yet the bedside assessment of these functions is compromised by patients' multiple impairments. Standardized behavioral scales such as the *Coma Recovery Scale-Revised* (CRS-R) have been developed to diagnose DoC, but there is also a need for neuropsychological measurement in these patients. The *Cognitive Assessment by Visual Election* (CAVE) was therefore recently created. In this study, we describe five patients in minimally conscious state (MCS) or emerging from the MCS (EMCS). Their cognitive profiles, derived from the CRS-R and CAVE, are presented alongside their neuroimaging results using structural magnetic resonance imaging (MRI) and fluorodeoxyglucose positron emission tomography (FDG-PET). Scores on the CAVE decreased along with the CRS-R total score, establishing a consistent behavioral/cognitive profile for each patient. Out of these five cases, the one with highest CRS-R and CAVE performance had the least extended cerebral hypometabolism. All patients showed structural and functional brain impairments that were consistent with their behavioral/cognitive profile as based on previous literature. For instance, the presence of visual and motor residual functions was respectively associated with a relative preservation of occipital and motor cortex/cerebellum metabolism. Moreover, residual language comprehension skills were found in the presence of preserved temporal and angular cortex metabolism. Some patients also presented structural impairment of hippocampus, suggesting the presence of memory impairments. Our results suggest that brain-behavior relationships might be observed even in severely brain-injured patients and they highlight the importance of developing new tools to assess residual cognition and language in MCS and EMCS patients. Indeed, a better characterization of their cognitive profile will be helpful in preparation of rehabilitation programs and daily routines.

## Introduction

After an acquired severe brain injury, patients generally go through a succession of altered states of consciousness: coma, unresponsive wakefulness syndrome (i.e., vegetative state—eye opening without signs of awareness) (1), minimally conscious state (MCS), and then emergence from the minimally conscious state (EMCS), when they are able to functionally communicate or use objects (2). Patients in a MCS have further been subcategorized in MCS *minus*, whose most frequent signs of consciousness are visual fixation and pursuit, automatic oriented motor reactions and localization to noxious stimulation (3), and in MCS *plus* patients who can also follow simple commands, intelligibly verbalize or intentionally communicate (4).

Previous literature has shown the importance of accurate diagnosis in DoC patients regarding daily management (i.e., pain treatment or stimulation protocols), end-of-life decisions and prognosis (5–7). Nevertheless, accurate diagnosis is challenging (8–13), with assessment being compromised by patients' multiple impairments, in particular motor skills and fluctuating arousal level (10, 11), as well as aphasia (14, 15) and impaired visual abilities (8). Several behavioral scales have been developed to assess patients' level of consciousness. Among them, the *Coma Recovery Scale-Revised* (CRS-R) (16) is currently considered the most sensitive validated diagnostic tool (17). There is still, however, a lack of standardized neuropsychological tests dedicated to the assessment of a wider range of cognitive functions in DoC patients. Indeed, although the CRS-R allows to precisely diagnose their levels of consciousness, patients' cognitive and language deficits cannot be specifically appreciated. Consequently, a new measure was recently developed on the grounds of clinical work: the *Cognitive Assessment by Visual Election* (CAVE) [(18); Murphy, unpublished thesis]. This assessment is based on the ability to understand language at a basic level and to visually fixate objects.

Due to the difficulty to behaviorally objectify signs of consciousness and cognition in this group of severely brain-injured patients, diverse neuroimaging techniques have been developed (19). A negative correlation was found between structural damage and the level of consciousness using voxel-based morphometry (VBM). The duration of a DoC has also been associated with larger brain lesions (20). Regarding functional brain imaging, active paradigms require preserved language functions and the ability to follow verbal commands, thus passive and resting state paradigms are more commonly used, either with positron emission tomography (PET) or magnetic resonance imaging (MRI) (21). Using fluorodeoxyglucose (FDG) PET, previous studies showed an association between consciousness recovery and the restoration of cerebral activity within a large frontoparietal network, comprised of two (internal and external) networks (22). The internal default mode network (DMN) encompasses the precuneus/posterior cingulate cortex, mesiofrontal/anterior cingulate cortex as well as the temporo-parietal junction, and is mainly dedicated to internal perception and self-awareness (23–25). The external lateral frontoparietal network is involved in executive control, external perception and environment awareness (22, 26). Finally, recent studies have shown that diverse neuroimaging and neurophysiology techniques tend to lead to compatible and consensual brain data in unresponsive and MCS patients (27–29), suggesting that it would be of benefit to combine these techniques to diagnose the DoC.

The presence of residual language and cognitive functions in DoC patients has been suggested by previous neuroimaging and electrophysiology studies (30–36). For example, residual cortical activity related to language processing was shown in two MCS patients, by comparing functional connectivity after listening to intelligible and unintelligible speech (37). To remedy the lack of cognitive behavioral measurement, Sergent and colleagues (38) used electroencephalography (EEG) and showed the advantages of a multidimensional cognitive evaluation based on low-level functions (i.e., own name recognition, temporal attention, spatial attention, detection of spatial incongruence and motor planning) and higher-level functions (i.e., modulations of previous effects by the global context) in detecting residual cognitive abilities in DoC patients.

In the present paper, we aim to study the behavioral and cognitive profile of five different patients in MCS and EMCS. Performance on the CRS-R and the CAVE were compared with their neuroimaging results using FDG-PET and structural MRI. By presenting these multiple cases, the importance of the development of new assessment tools such as the CAVE to refine the cognitive profile of MCS and EMCS patients, is emphasized. Specifically, it is hypothesized that there is an association between patients' structural and functional brain damage and their behavioral/cognitive profile, consistent with previous studies establishing neural correlates of behavior, language and cognition.

## Materials and methods

This prospective study includes five patients who were consecutively recruited at the University Hospital of Liège. All patients completed a battery of behavioral tests and neuroimaging assessments during a one-week hospitalization, based on clinical demand. Patients with absence of visual pursuit or visual evoked potentials (as observed by an experimented ophthalmologist) were excluded, as some functional vision is required to perform the CAVE. The control group consisted of 58 healthy subjects as controls for FDG-PET (34/58) and MRI data (36/58). The study was approved by the Ethics Committee of the Faculty of Medicine of the University of Liege and written informed consents, including for publication of data, were obtained from the patients' legal representatives and from the healthy control subjects.

### Bedside behavioral assessments

#### Coma recovery scale-revised (CRS-R)

The CRS-R was used for clinical diagnosis. This scale includes 23 items divided in 6 sub-scales: auditory, visual, motor, oro-motor/verbal, communication, and arousal, each assessing different items of increasing complexity (16). Some of the items are diagnostic criteria for MCS (e.g., visual pursuit, automatic oriented motor reactions, or response to command) and EMCS (i.e., functional communication and/or use of objects), and the total score ranges from 0 to 23. Following the most recent guidelines to reduce misdiagnosis (39), at least five clinical assessments within a short time interval (i.e., 1 week) were conducted. The highest CRS-R score and diagnostic category of the week was retained for final diagnosis.

#### Cognitive assessment by visual election (CAVE)

The CAVE includes 6 sub-tests to evaluate the recognition of real objects, numbers, written words, letters, pictures, and colors (18). Each of these sub-tests contains 10 items (Supplementary Material I), with a cut-off score of 8/10 based on binomial distribution. A target object is presented simultaneously with a distractor (e.g., a pen on the left and a fork on the right visual field) and the patient is asked to look at the target (e.g., “look at the pen”). As this test requires at least the preservation of visual fixation, this tool is dedicated to MCS *minus*, MCS *plus*, and EMCS patients. It usually takes between 10 and 30 min to administer, depending on the ability to objectify patient's eye fixations and patient's fatigue. The scoring sheet is presented in Supplementary Material I. An extended version of the CAVE proposes additional subtests, including a visual memory recognition exercise that was attempted with our patients (except case 4). First, patients were presented five pictures (one at a time) and asked to memorize them. Afterward, each target was presented with a distractor and they were asked to look at the previously shown picture.

### Electrophysiological measurement

A clinical EEG was performed using 19 electrodes and interpreted by a certified neurologist to assess the severity of the encephalopathy.

### MRI

MRI data was acquired using a 3 Tesla scanner (Siemens Trio, Siemens Medical Solutions, Erlangen, Germany). Structural MRI data were obtained with T1-weighted 3D gradient echo images using 120 slices (repetition time = 2300 ms, echo time = 2.47 ms, voxel size = 1 × 1 × 1.2 mm^3^, flip angle = 9°, field of view = 256 × 256 mm^2^).

#### Voxel-based morphometry (VBM)

A T1 voxel-based morphometry analysis of brain structure using the VBM8 toolbox (Structural Brain Mapping Group, Christian Gaser, Department of Psychiatry, University of Jena, Germany) was carried out. T1 MRI images were segmented into gray and white matter and cerebrospinal fluid using the unified segmentation module (40). These segmented gray and white matter images were used to obtain a more accurate registration model using DARTEL (41, 42). The images of each participant were then normalized into the DARTEL template in MNI space. The gray matter images were modulated to ensure the preservation of their volumes after the normalization step. The modulated normalized gray matter images were smoothed with a Gaussian isotropic kernel of 12 mm of full width at half maximum (FWHM). The differences in gray matter volume were investigated by comparing each patient with a group of 36 healthy control subjects (mean age = 46 ± 16 years old, 13 women) using a parametric two-sample *t*-test. Both the total intracranial volume and age, centered to mean and standardized to 1, were then used as covariates. Results were considered significant at family-wise error (FWE) corrected *p* < 0.05 at cluster level and cluster defining threshold *p* < 0.001.

### FDG-PET

A resting 18F-FDG PET/CT scan was performed after intravenous injection of ~150 MBq of FDG using a Gemini TF PET-CT scanner (Philips Medical Systems) as described elsewhere (43). The scan started 30 min after an intravenous injection of the tracer and the scan duration was 12 min. FDG-PET images for each patient were manually reoriented using SPM12. The images were then spatially normalized, smoothed (with a 14 mm FWHM Gaussian filter) and analyzed. Patient data were compared to 34 healthy control subjects (age range 19–70 years, 15 women). SPM analysis identified brain regions with decreased and relatively preserved metabolism in each patient compared to healthy control subjects (global normalization was performed by proportional scaling). The resulting set of voxel values for each contrast, constituting a statistical parametric map of the *t*-statistics (SPM{*t*}), was transformed to the unit normal distribution (SPM{Z}) and thresholded at voxel-wise *p* < 0.05 FWE-corrected and at *p* < 0.001 uncorrected.

## Results

**Figure 1:**
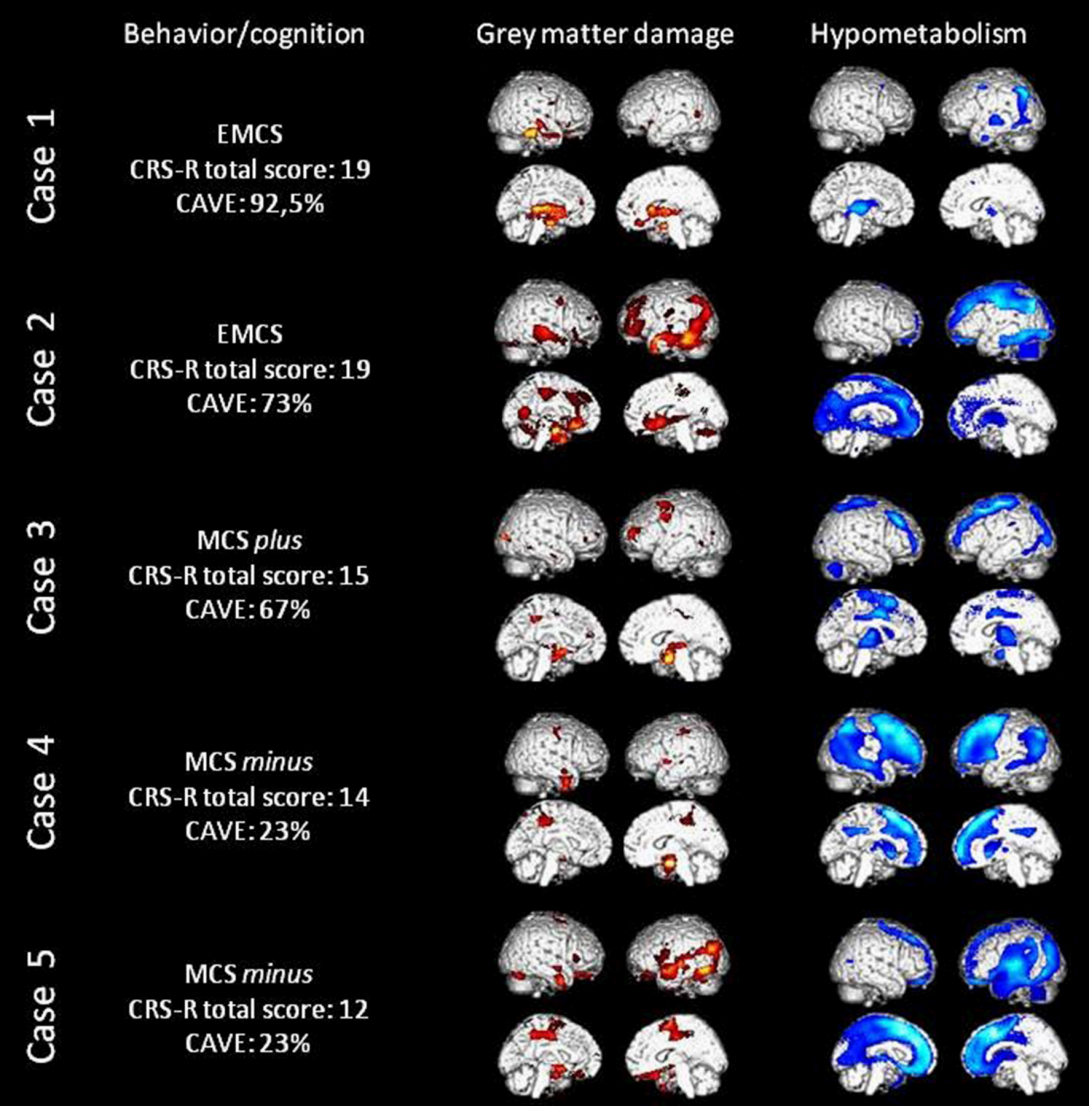
Behavioral and cognitive data, loss of gray matter volume (in red) as assessed with MRI voxel-based morphometry and cerebral hypometabolism (in blue) as assessed with FDG-PET in all five patients. Here the threshold is uncorrected 0.001 for display values (please refer to Table 2 for corrected results).

The main results of the five patients (all right-handed; age range: 20–66 years old; one woman) are presented in Figure 1. The CRS-R and CAVE scores are presented in Table 1. All VBM and PET statistical results are presented in Table 2 (most significant data) and S1 (Supplementary Material II). The significant regions' names were derived from the AAL2 atlas, using *bspmview* tool (http://www.bobspunt.com/bspmview/, doi: 10.5281/zenodo.168074).

**Table 1:** Behavioral scores at the CRS-R and the CAVE.

		**Case 1**	**Case 2**	**Case 3**	**Case 4**	**Case 5**
CRS-R	Final diagnosis	EMCS	EMCS	MCS+	MCS-	MCS-
	Auditory score	4^*^	4^*^	3^*^	2	1
	Visual score	5^*^	4^*^	3^*^	3^*^	3^*^
	Motor score	5^*^	6^#^	5^*^	5^*^	5^*^
	Oromotor/verbal score	1	2	2	2	1
	Communication score	2^#^	1^*^	0	0	0
	Arousal score	2	2	2	2	2
	Total score	19	19	15	14	12
CAVE	Real objects	9/10	*7/10*	10/10	*4/10*	*4/10*
	Numbers	9/10	9/10	8/10	NA	*3/10*
	Words	9/10	*6/10*	*1/10*	*2/10*	*1/10*
	Letters	10/10	*5/10*	*7/10*	NA	*1/10*
	Pictures	NA	10/10	9/10	NA	*3/10*
	Colors	NA	*7/10*	*5/10*	NA	*2/10*
	Percentage of success	92.5%	73%	67%	23%	23%
	Memory	5/5	3/5	1/5	NA	1/5
	Left/right differences	No	Yes	No	No	Yes

# *indicates emergence of minimally conscious state (EMCS)*,

* *indicates MCS*.

*The CAVE scores in italics are below the cut-off score and thus considered as failed. NA, not administered*.

**Table 2:** Regions showing significant gray matter hypotrophy, impaired and preserved metabolism.

	**Brain regions**	***p*(FWE-corr)**	***T***	***x***	***y***	***z***
**GRAY MATTER**						
Case 1 < CTR	L hippocampus	0	6,4	−30	−15	−17
Case 2 < CTR	L fusiform cortex	0	11,5	−29	−15	−24
	L medial orbitofrontal cortex	0	8,1	−8	27	−12
	R superior temporal cortex	0,002	6,1	68	−9	−9
	L calcarine	0,035	4,9	−11	−60	11
	R cerebellum	0,038	4,3	23	−77	−30
Case 3 < CTR	R hippocampus	0,004	5,9	20	−6	−20
	L precentral cortex	0,025	4,7	−27	−4	53
	L hippocampus	0,036	4,7	−15	−6	−12
Case 4 < CTR	R amygdala	0	6,5	30	−4	−20
Case 5 < CTR	L inferior temporal cortex	0	8,0	−53	−69	−9
	R supplementary motor area	0,001	5,2	11	−1	65
**HYPOMETABOLISM**						
Case 1 < CTR	L angular gyrus	0,016	5,3	−46	−70	38
	L thalamus	0,015	5,2	−8	−18	6
Case 2 < CTR	L inferior parietal	0	15,6	−54	−26	36
Case 3 < CTR	L precentral cortex	0	12,2	−28	−18	68
	R middle frontal cortex	0,003	9,2	34	34	38
	R precentral cortex	0,012	6,0	26	−28	70
	L middle occipital cortex	0,006	5,6	−32	−90	8
	Brain stem	0,002	5,5	2	−24	−4
Case 4 < CTR	R middle frontal cortex	0	8,5	44	10	50
	L caudate	0,013	7,1	−16	12	8
	L middle temporal cortex	0	6,5	−50	−68	18
	R middle cingulate cortex	0,02	4,7	4	−50	34
Case 5 < CTR	L middle temporal cortex	0	15,4	−54	−58	20
**PRESERVED METABOLISM**						
Case 1 > CTR	R frontal lobe (white matter)	0	7,2	26	24	24
	R angular gyrus	0,041	4,5	48	−48	32
Case 2 > CTR	R amygdala	0	13,1	34	2	−24
Case 3 > CTR	R frontal lobe (white matter)	0	9,1	46	−2	18
	L supramarginal gryus	0	9,1	−50	−28	30
Case 4 > CTR	L insula	0	10,0	−30	−8	18
	R insula	0,006	9,8	32	−4	18
	R cerebellum	0	7,0	20	−56	−20
Case 5 > CTR	R amygdala	0	17,3	34	0	−28

*L, left; R, right, CTR, healthy control subjects*.

### Case 1

This patient was admitted to our hospital 16 months after a traumatic brain injury. He was diagnosed as EMCS (with a total CRS-R score of 19/23) because of his ability to functionally communicate using “yes” and “no” cards. Due to fatigue and time limitation, only four CAVE sub-tests were administered. According to the cut-off score, he was able to recognize objects, numbers, written words and letters, as well as to memorize five pictures (Table 1). Overall, case 1 correctly responded to 92.5% of the administered items of the CAVE.

The clinical EEG showed abnormalities regarding the posterior and temporal derivations of the left hemisphere. As seen in Table 2 and Figure 1, the VBM shows gray matter damage in the left hippocampus. PET hypometabolism was observed in the left thalamus and angular gyrus (*p* < 0.05 FWE corrected), as well as the left putamen and part of the left inferior and middle temporal gyrus, the left precentral cortex and the right superior frontal cortex (*p* < 0.001 uncorrected). The most preserved metabolism was shown in the right angular gyrus (*p* < 0.05 FWE corrected) and in the right insula, middle frontal cortex, post-central cortex, rolandic operculum and superior temporal cortex (*p* < 0.001 uncorrected).

### Case 2

Case 2 had a stroke and epilepsy due to post-surgery complications 30 months before his admission to our hospital. He was diagnosed as EMCS (with a total CRS-R score of 19/23), as he was able to functionally use objects but not to functionally communicate. Using the CAVE, the patient showed a good performance in recognizing numbers and pictures (Table 1). He was just below the cut-off score with real objects and colors but he had more difficulties with discriminating letters and written words and in memorizing the pictures. Unilateral spatial neglect was suspected since his performance was better when the target item was presented on his left side. Case 2 performed well for 73% of administered items.

The clinical EEG suggests significant left hemispheric damage with a nascent encephalopathy. Neuroimaging results also show left hemisphere structural and functional damage. Significant hypotrophy in the left fusiform, left medial orbitofrontal and right superior temporal cortices was noted, as well as in the left calcarine sulcus and right cerebellum. Hypometabolism was also observed in the left inferior parietal cortex (*p* < 0.05 FWE corrected) and in the left supplementary motor area, superior frontal cortex, cingulate cortex, precuneus, fusiform cortex, superior parietal cortex, hippocampus and amygdala, as well as bilateral rectus gyri and thalami (*p* < 0.001 uncorrected). The regions showing the most preserved metabolism were the right amygdala (*p* < 0.05 FWE corrected) and the bilateral cerebellum and right middle frontal cortex, temporal, parietal and occipital lobules (*p* < 0.001 uncorrected).

### Case 3

This patient came to our hospital 13 months after a traumatic brain injury. The diagnosis was MCS *plus* (with a total CRS-R score of 15/23) since he was able to follow simple verbal commands (e.g., “Look up,” “Turn your head” and “Close your eyes”). His cognition was more impaired than case 1 and qualitatively very different from case 2 (Table 1). He could perform some sub-tests, namely recognizing real objects, numbers and pictures. The other attempted sub-tests (including memory) led to performance lower than the cut-off score. This patient successfully responded to 67% of presented items.

The clinical EEG was biased by abundant movement artifacts. Structural damage was shown using VBM in the bilateral hippocampi and in the right precentral cortex. The PET analysis showed significant hypometabolism in bilateral precentral cortex, right middle frontal cortex, and left middle occipital cortex (*p* < 0.05 FWE corrected), as well as in the left inferior occipital cortex, middle frontal gyrus and supplementary motor area and bilateral middle cingulate cortex and thalami (*p* < 0.001 uncorrected). The most preserved metabolism was observed in the left supramarginal gyrus (*p* < 0.05 FWE corrected) and the right inferior frontal, inferior parietal, angular, and superior temporal cortex, as well as left inferior frontal, middle and superior temporal cortex (*p* < 0.001 uncorrected).

### Case 4

Case 4 sustained a hypoxic-ischemic brain injury following an insulin overdose; she was 3 years post-hypoglycemia. This patient showed the requested visual functions, as well as automatic oriented motor reactions, therefore she was considered as being in a MCS *minus* with a CRS-R total score of 14/23. Nevertheless, she was an atypical MCS *minus* patient due to her ability to walk when guided by someone else. Using the CAVE, she failed to recognize real objects, numbers, words and colors. The remaining subtests (i.e., letters and pictures recognition) were not administered due to patient fatigue. Case 4 performed well for 23% of the administered items.

Despite the presence of muscular artifacts, the clinical EEG showed significant encephalopathy with no sign of lateralization. The neuroimaging data showed hypotrophy of the right amygdala. Moreover, hypometabolism was mainly found in the right middle frontal and cingulate cortex and in the left caudate and middle temporal cortex (*p* < 0.05 FWE corrected), as well as in bilateral angular gyrus, caudate, putamen, thalami, and frontal cortex, in the right middle temporal and inferior parietal cortex, in the left insula and middle temporal cortex (*p* < 0.001 uncorrected). On the contrary, the most preserved metabolism was shown in the right cerebellum (*p* < 0.05 FWE corrected), in the bilateral insula and putamen, and in the left cerebellum, precuneus, paracentral and postcentral cortex (*p* < 0.001 uncorrected).

### Case 5

This last patient had a stroke 13 months before his stay in our hospital. He was diagnosed as MCS *minus* with a CRS-R total score of 12/23. He did not show any residual language ability but he was able to visually fixate and track objects, as well as to automatically open his mouth when a spoon was moved toward it (i.e., automatic motor response). Similarly to case 4, this patient failed to recognize (and memorize) the visual targets, despite his high arousal enabling us to attempt all CAVE subtests. As for case 4, case 5 visually fixed the target item for 23% of the trials but left/right differences were observed.

The clinical EEG showed a symmetrical slow dysrythmia with no paroxysm. Gray matter hypotrophy was shown in the left inferior temporal cortex and right supplementary motor area. PET results show the presence of significant hypometabolism in the left middle temporal cortex (*p* < 0.05 FWE corrected), the bilateral superior frontal and cingulate cortex, the left thalamus, precuneus, and parietal cortex (*p* < 0.001 uncorrected). Preserved metabolism in the right amygdala was observed (*p* < 0.05 FWE corrected), as well as in the vermis, the bilateral cerebellum, the left hippocampus and the right parieto-occipito-temporal regions including the right precuneus and angular gyrus (*p* < 0.001 uncorrected).

## Discussion

In this study, patients in MCS or EMCS have been assessed with a broad spectrum of (para)clinical tools. Using the CAVE, it has been possible to evaluate the cognitive profile of severely brain-injured patients, and the importance of the use of such new bedside neuropsychological assessments is highlighted. It was hypothesized that CAVE profiles would correspond to patients' cerebral structure and brain activity. Comparing all patients, the highest scorer on bedside behavioral and language-based cognitive assessments (i.e., case 1) showed less extended levels of cerebral hypometabolism. It was also found that the percentage of success on the CAVE decreased along with the CRS-R total score (see Table 1), establishing a consistent behavioral/cognitive profile for each patient. The cognitive profile obtained from the CRS-R and the CAVE was mostly found to correspond to structural and functional results. As shown in Figure 1, both neuroimaging techniques also seem in agreement: gray matter damages are generally paralleled with hypometabolism of the same structures, and this hypometabolism is even more widespread. Below, we discuss cognitive functions in different domains and compare the behavioral results with neuroimaging findings.

### Visual functions

All patients were able to visually fixate and pursuit objects and all showed a relative structural and metabolic preservation of occipital lobule. Regarding case 1, the ability to visually fixate objects and the use of a visually-based communication code were consistent with the absence of significant hypometabolism and gray matter hypotrophy in the occipital cortex. The difficulty to perform well with letters, words, and colors in case 3 may be consistent with the apparent hypometabolism within the left occipital cortex (44–47). In addition, number recognition appeared intact in this patient. This ability has been shown to rely on the right lateral occipital area (48), and our patient showed no significant hypometabolism in this area. Hence, our findings suggest a dissociation between letters and numbers recognition which was associated with specific occipital lesions. Both MCS *minus* patients were unable to successfully recognize the CAVE target items. Despite their ability to visually fixate one object when it was presented alone, none of these two patients showed responses to command, which suggest that they did not understand the task instructions (see next section).

Unilateral spatial neglect and/or hemianopia were suspected in case 2 and case 5 since there was a significant difference in the performance between left and right CAVE target items. Indeed, a deviation of their eyes toward their left side was noted in both patients. Karnath and coworkers have highlighted the role of a perisylvian network in spatial neglect (49), including the temporo-parietal junction, the temporal lobules and underlying insula, as well as the ventro-lateral prefrontal cortex. Accordingly, these two patients showed hypometabolism and hypotrophy of gray matter in some of these cerebral regions.

### Language and executive functions

Case 1 was the only patient who could functionally communicate using a “yes”/“no” code. This ability requires language and executive functions such as mental flexibility. Hence recovery of communication does not seem surprising due to the preserved metabolism and absence of gray matter damage in frontal lobules (50, 51). Besides communication, this patient was also able to follow simple commands and to understand the “look at” commands during the administration of the CAVE. Nevertheless, the EEG and PET analysis reported abnormalities regarding the posterior and temporal derivations of the left hemisphere, shown to be dedicated to semantics (52). Specifically, we found peaks of hypometabolism within the left angular gyrus, which was related to sentence comprehension (52–54). Still, this patient's residual language skills may emerge from neural plasticity using the cerebral areas that are either around the lesion, or in the contralateral cerebral regions (55–59). Indeed, right angular gyrus and superior temporal cortex showed preserved metabolism.

In contrast, case 2 was unable to functionally communicate and read written letters and words during the CAVE assessment. This was consistent with the massive left cerebral lesion that was detected with VBM, PET and clinical EEG (52, 60). More precisely, this patient showed hypometabolism and gray matter reduction in the left fusiform cortex, known to be the “visual word form area” (54, 60, 61). Therefore, these data matched well with his inability to recognize letters and words. Taken together, the CAVE results suggested that the more linguistic were the items, the more difficult it was for this patient to answer. Thus, it is likely that this patient had severe aphasic difficulties. Nevertheless, he was systematically able to follow (and thus understand) commands. This may correspond with the absence of hypometabolism in areas such as the left superior temporal cortex (52). In addition, similarly to case 1 it could be argued that he recovered such abilities by means of neural plasticity.

Case 3 was able to understand and follow commands and he could recognize objects, pictures and numbers. All these skills require residual language comprehension and relative preservation of semantic processing, which is related to left temporal areas (52). Accordingly, we observed the absence of gray matter hypotrophy and the presence of preserved metabolism regarding the left temporal lobule. Again, this patient showed an inability to recognize letters and written words. If this patient, contrary to case 2, did not show impairment of the left fusiform gyrus (i.e., the visual word form area), he still showed hypometabolism in regions that are very close (i.e., the left inferior and middle occipital cortex). These findings were also consistent with the patient's inability to discriminate different colors (44, 62).

The inability of case 4 and case 5 to show language-based signs of consciousness (i.e., command-following, intelligible verbalization and communication) and to recognize CAVE items corresponded to their hypometabolism, notably regarding the left angular gyrus (52). These results implied a lack of verbal comprehension due to accumulated language and cognitive impairments. Indeed, more impaired language functions in MCS *minus* than in MCS *plus* patients was suggested by previous studies (63, 64).

### Motor functions

Repeated assessments on the CRS-R did not demonstrate functional use of objects in case 1 but it was noted that this patient tended to grab his bed sheets and try to reach objects. Accordingly, we did not observe hypometabolism within the motor cerebral areas (Figure 1). Furthermore, case 2's ability to functionally use some objects (i.e., a comb) could emerge from preserved right motor areas. Our third and fifth cases obtained the same motor subscale score at the CRS-R as case 1 since they showed automatic oriented movements with their mouth. The inability to move their limbs could thus be related to case 3's hypometabolism of the precentral cortex and supplementary motor area and to case 5's damage of the right supplementary motor area. Interestingly, case 4 was an atypical MCS *minus* patient because she was able to walk despite her inability to respond to commands. This capacity was probably possible because of preserved metabolism of the left paracentral and postcentral sensorimotor cortex (65–67). In addition, this patient also showed a preserved cerebellum and previous studies have highlighted its role in gait and movement coordination (68).

### Memory and consciousness

Case 1 performed perfectly to the memory subtest. Nevertheless, it is a recognition task and other higher order memory processes might still be impaired. Indeed, case 1 (as well as case 3) showed impaired gray matter structure in the hippocampus, which has shown to be related to episodic memory in numerous previous studies [e.g., (69, 70)]. Since memory difficulties were presented on this subtest by the other cases, one could thus hypothesize the presence of memory impairment in all five patients.

All patients were no less than minimally conscious, and at least part of the external frontoparietal network was preserved in all of them (22). Interestingly, in our atypical case 4 the metabolism of this external network seemed less preserved than the other cases. The internal DMN was probably slightly more affected than external network in our patients. For instance, the left precuneus was shown to be hypometabolic in case 2 and case 5, whereas the left temporo-parietal junctions (also involved in the DMN) seem hypometabolic in four patients. Lastly, the thalamus, known to play an important role in consciousness (71), was hypometabolic in all five patients. Since thalamo-cortical alterations were found in other brain-injured patients with chronic fatigue problems (72), case 1's fatigue might also be at least partially explained by the left thalamus functional impairment.

### Limitations

This multiple case report only provides preliminary findings; more patients are needed in order to overcome statistical limitations and confirm the relationships between cognition and brain structure and function at the group level. The heterogeneity of DoC patients makes this research very challenging. Moreover, the performance at the CAVE is multi-determined, requiring visual functions, language comprehension and other subtest-specific abilities such as reading. As such, the CAVE might allow us to detect the presence of aphasia in our patients, but it does not discriminate or specify which language functions are altered (e.g., phonology vs. semantics). New material could be included to evaluate MCS and EMCS patients' cognitive functions in a more specific way. Finally, the CAVE seems to be useful only for patients who are at least MCS *plus*.

### Conclusion

In this study, the performance of all patients using the CRS-R and the CAVE was consistent, and it mostly corresponded to their brain structure and metabolism in line with previous research on patients with focal cerebral lesions. For instance, the ability to recognize visually presented objects, or items based on their name was linked to a relative preservation of visual and language metabolism. In addition, residual language comprehension skills were found in the presence of preserved temporal and angular cortex metabolism. These results suggest that brain-behavior relationships might be observed even in severely brain-injured patients. This research further highlights the importance of the development of behavioral assessment tools, such as the CAVE, both to inform clinical practice and for scientific interest. Clinically, besides the CRS-R this new test allows to refine the patient's cognitive profile. This knowledge will be helpful in preparation of rehabilitation programs and daily routines. Such information may be important also for the investigation of the neural correlates of behavior and cognition in patients with severe brain injury.

## Author contributions

CA, LM, and OG conceived and planned the presented research. LM previously created the CAVE. CA, OG, MB, SKL, AT, and SW contributed to data analyses and interpretation. CA, HC, JA, and MC acquired the data. CA drafted the manuscript under OG's supervision and all authors provided critical feedback and helped shape the manuscript.

### Conflict of interest statement

The authors declare that the research was conducted in the absence of any commercial or financial relationships that could be construed as a potential conflict of interest.
