# Supplementary material for: Brain, Behavior, and Cognitive Interplay in Disorders of Consciousness: A Multiple Case Study
Source: Front Neurol. 2018 Aug 14;9:665. doi: 10.3389/fneur.2018.00665 (PMC6103268; doi:10.3389/fneur.2018.00665)
Supplement: Supplementary file 1 [file Data_Sheet_1.docx]

**Brain, behavior and cognitive interplay in disorders of consciousness:**

**A multiple case study**

**Charlène Aubinet¹*, Lesley Murphy², Mohamed Ali Bahri³, Stephen Karl Larroque¹, Helena Cassol¹, Jitka Annen¹, Manon Carrière¹, Sarah Wannez¹, Aurore Thibaut¹, Steven Laureys¹, Olivia Gosseries¹**

**SUPPLEMENTARY MATERIAL**

Supplementary material I – CAVE scoring sheet

Supplementary material II – Significant clusters emerging from the analyses

**Supplementary material I – CAVE scoring sheet**

**
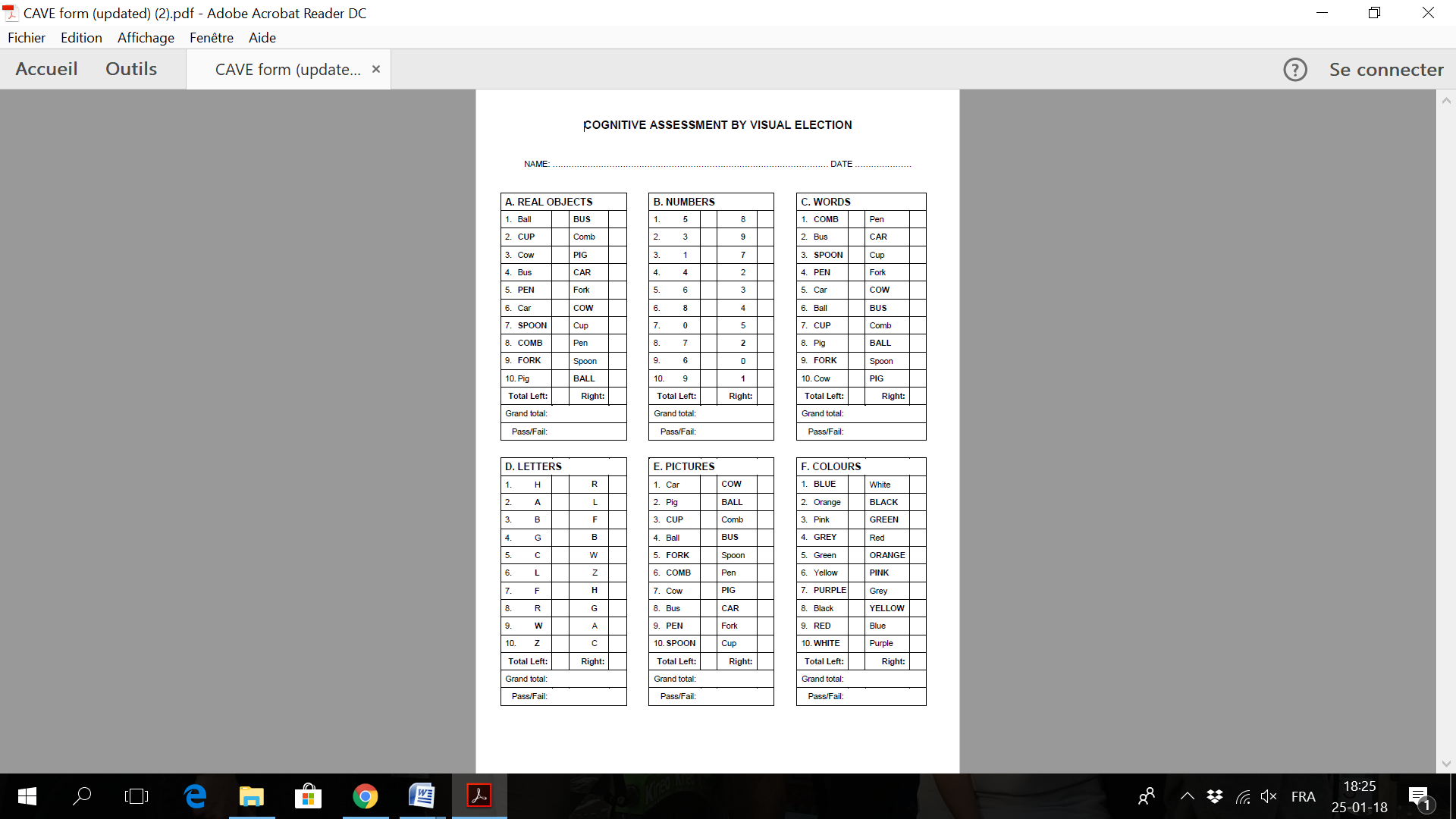
**

**Supplementary material II – Significant clusters**

Table S1: Significant clusters emerging after the VBM (grey matter) and PET analyses.

| **Case** | **Analysis** | **Set** | | **Cluster** | | | | **Peak** | | | | **Coordinates** | | | |
| --- | --- | --- | --- | --- | --- | --- | --- | --- | --- | --- | --- | --- | --- | --- | --- |
|  |  | *p* | *c* | *p*(FWE-corr) | *p*(FDR-corr) | equiv *k* | *p*(unc) | *p*(FWE-corr) | *p*(FDR-corr) | *T* | equiv *Z* | *x* | *y* | *z* |  |
| **Case 1** | Grey matter reduction | 0,001 | 9 | 0,000 | 0,000 | 18041 | 0,000 | 0,002 | 0,034 | 6,393 | 5,120 | -30 | -15 | -17 |  |
|  | Hypometabolism | 0,000 | 9 | 0,016 | 0,033 | 1115 | 0,007 | 0,030 | 0,125 | 5,300 | 4,462 | -46 | -70 | 38 |  |
|  |  |  |  | 0,015 | 0,033 | 1137 | 0,007 | 0,038 | 0,125 | 5,200 | 4,395 | -8 | -18 | 6 |  |
|  |  |  |  | 0,606 | 0,550 | 80 | 0,428 | 0,164 | 0,336 | 4,500 | 3,965 | 26 | 18 | 50 |  |
|  |  |  |  | 0,247 | 0,390 | 298 | 0,130 | 0,294 | 0,336 | 4,200 | 3,757 | -62 | -24 | -6 |  |
|  |  |  |  | 0,559 | 0,550 | 99 | 0,376 | 0,300 | 0,336 | 4,200 | 3,750 | -48 | -6 | -34 |  |
|  |  |  |  | 0,550 | 0,550 | 103 | 0,366 | 0,369 | 0,370 | 4,100 | 3,666 | -30 | -12 | 0 |  |
|  |  |  |  | 0,540 | 0,550 | 107 | 0,357 | 0,441 | 0,416 | 4,000 | 3,588 | -48 | 0 | 52 |  |
|  |  |  |  | 0,800 | 0,832 | 17 | 0,739 | 0,752 | 0,689 | 3,600 | 3,270 | -32 | -88 | 28 |  |
|  |  |  |  | 0,851 | 0,875 | 5 | 0,875 | 0,879 | 0,971 | 3,400 | 3,103 | 12 | -90 | 8 |  |
|  |  |  |  | 0,801 | 0,850 | 14 | 0,773 | 0,771 | 0,790 | 3,400 | 3,229 | 34 | -18 | -20 |  |
|  |  |  |  | 0,857 | 0,932 | 2 | 0,932 | 0,866 | 0,962 | 3,300 | 3,106 | -6 | -54 | 22 |  |
|  | Preserved metabolism | 0,024 | 6 | 0,000 | 0,000 | 3392 | 0,000 | 0,000 | 0,002 | 7,200 | 5,534 | 26 | 24 | 24 |  |
|  |  |  |  | 0,233 | 0,243 | 313 | 0,122 | 0,141 | 0,219 | 4,600 | 4,013 | -22 | 46 | -4 |  |
|  |  |  |  | 0,041 | 0,058 | 803 | 0,019 | 0,172 | 0,219 | 4,500 | 3,948 | 48 | -48 | 32 |  |
|  |  |  |  | 0,336 | 0,282 | 221 | 0,188 | 0,247 | 0,219 | 4,300 | 3,823 | 36 | 2 | -42 |  |
|  |  |  |  | 0,488 | 0,369 | 131 | 0,308 | 0,304 | 0,222 | 4,200 | 3,745 | -34 | 32 | 20 |  |
|  |  |  |  | 0,554 | 0,371 | 101 | 0,371 | 0,583 | 0,428 | 3,800 | 3,445 | 20 | -54 | 54 |  |
| **Case 2** | Grey matter reduction | 0,000 | 18 | 0,000 | 0,000 | 27549 | 0,000 | 0,000 | 0,000 | 11,546 | 7,258 | -29 | -15 | -24 |  |
|  |  |  |  | 0,000 | 0,000 | 11630 | 0,000 | 0,000 | 0,000 | 8,075 | 5,958 | -8 | 27 | -12 |  |
|  |  |  |  | 0,002 | 0,004 | 3881 | 0,001 | 0,005 | 0,006 | 6,073 | 4,941 | 68 | -9 | -9 |  |
|  |  |  |  | 0,039 | 0,046 | 1634 | 0,015 | 0,027 | 0,022 | 5,369 | 4,519 | -11 | -26 | 36 |  |
|  |  |  |  | 0,035 | 0,046 | 1701 | 0,014 | 0,090 | 0,058 | 4,857 | 4,188 | -11 | -60 | 11 |  |
|  |  |  |  | 0,038 | 0,046 | 1657 | 0,015 | 0,280 | 0,161 | 4,326 | 3,822 | 23 | -77 | -30 |  |
|  | Hypometabolism | 0,082 | 4 | 0,000 | 0,000 | 23919 | 0,000 | 0,000 | 0,000 | 15,600 | 65535,000 | -54 | -26 | 36 |  |
|  |  |  |  | 0,671 | 0,958 | 26 | 0,684 | 0,455 | 0,382 | 3,800 | 3,466 | 32 | 6 | -46 |  |
|  |  |  |  | 0,709 | 0,958 | 16 | 0,760 | 0,658 | 0,665 | 3,600 | 3,256 | -2 | -36 | -50 |  |
|  |  |  |  | 0,789 | 0,958 | 1 | 0,958 | 0,801 | 0,995 | 3,400 | 3,093 | 4 | 24 | 18 |  |
|  | Preserved metabolism | 0,803 | 1 | 0,000 | 0,000 | 62424 | 0,000 | 0,000 | 0,000 | 13,100 | 7,703 | 34 | 2 | -24 |  |
| **Case 3** | Grey matter reduction | 0,000 | 18 | 0,004 | 0,030 | 3129 | 0,002 | 0,007 | 0,118 | 5,919 | 4,852 | 20 | -6 | -20 |  |
|  |  |  |  | 0,025 | 0,086 | 1916 | 0,010 | 0,120 | 0,481 | 4,737 | 4,107 | -27 | -4 | 53 |  |
|  |  |  |  | 0,036 | 0,086 | 1687 | 0,014 | 0,124 | 0,481 | 4,722 | 4,098 | -15 | -6 | -12 |  |
|  | Hypometabolism | 0,000 | 10 | 0,000 | 0,000 | 7400 | 0,000 | 0,000 | 0,000 | 12,200 | 7,465 | -28 | -18 | 68 |  |
|  |  |  |  | 0,003 | 0,004 | 1772 | 0,001 | 0,000 | 0,000 | 9,200 | 6,429 | 34 | 34 | 38 |  |
|  |  |  |  | 0,109 | 0,088 | 515 | 0,053 | 0,002 | 0,004 | 6,400 | 5,117 | 26 | -8 | -28 |  |
|  |  |  |  | 0,012 | 0,011 | 1208 | 0,006 | 0,005 | 0,009 | 6,000 | 4,886 | 26 | -28 | 70 |  |
|  |  |  |  | 0,006 | 0,007 | 1484 | 0,003 | 0,012 | 0,018 | 5,600 | 4,691 | -32 | -90 | 8 |  |
|  |  |  |  | 0,002 | 0,004 | 1822 | 0,001 | 0,015 | 0,021 | 5,500 | 4,631 | 2 | -24 | -4 |  |
|  |  |  |  | 0,543 | 0,452 | 105 | 0,362 | 0,207 | 0,147 | 4,400 | 3,884 | -14 | 14 | 12 |  |
|  |  |  |  | 0,163 | 0,117 | 405 | 0,082 | 0,384 | 0,274 | 4,100 | 3,647 | 36 | -70 | -34 |  |
|  |  |  |  | 0,791 | 0,757 | 19 | 0,722 | 0,653 | 0,538 | 3,700 | 3,372 | -66 | -34 | 34 |  |
|  |  |  |  | 0,806 | 0,757 | 15 | 0,757 | 0,798 | 0,763 | 3,500 | 3,214 | 36 | -88 | 8 |  |
|  | Preserved metabolism | 0,023 | 6 | 0,000 | 0,000 | 7051 | 0,000 | 0,000 | 0,000 | 9,100 | 6,399 | 46 | -2 | 18 |  |
|  |  |  |  | 0,000 | 0,000 | 5503 | 0,000 | 0,000 | 0,000 | 9,100 | 6,379 | -50 | -28 | 30 |  |
|  |  |  |  | 0,199 | 0,205 | 352 | 0,102 | 0,244 | 0,199 | 4,300 | 3,825 | -38 | 2 | -32 |  |
|  |  |  |  | 0,410 | 0,365 | 172 | 0,243 | 0,358 | 0,222 | 4,100 | 3,676 | 12 | 36 | 36 |  |
|  |  |  |  | 0,628 | 0,548 | 71 | 0,457 | 0,360 | 0,222 | 4,100 | 3,674 | -14 | 36 | 2 |  |
|  |  |  |  | 0,794 | 0,731 | 18 | 0,731 | 0,754 | 0,648 | 3,600 | 3,265 | 16 | 48 | -12 |  |
| **Case 4** | Grey matter reduction | 0,000 | 18 | 0,000 | 0,003 | 4948 | 0,000 | 0,001 | 0,017 | 6,543 | 5,202 | 30 | -4 | -20 |  |
|  | Hypometabolism | 0,023 | 6 | 0,000 | 0,000 | 31770 | 0,000 | 0,000 | 0,000 | 8,500 | 6,138 | 44 | 10 | 50 |  |
|  |  |  |  | 0,013 | 0,012 | 1182 | 0,006 | 0,000 | 0,002 | 7,100 | 5,475 | -16 | 12 | 8 |  |
|  |  |  |  | 0,000 | 0,000 | 2838 | 0,000 | 0,001 | 0,003 | 6,500 | 5,195 | -50 | -68 | 18 |  |
|  |  |  |  | 0,126 | 0,075 | 472 | 0,062 | 0,011 | 0,009 | 5,700 | 4,713 | 36 | -20 | -20 |  |
|  |  |  |  | 0,020 | 0,014 | 1024 | 0,010 | 0,104 | 0,065 | 4,700 | 4,107 | 4 | -50 | 34 |  |
|  |  |  |  | 0,443 | 0,271 | 152 | 0,271 | 0,234 | 0,139 | 4,400 | 3,840 | -34 | -20 | -18 |  |
|  | Preserved metabolism | 0,000 | 13 | 0,000 | 0,000 | 3637 | 0,000 | 0,000 | 0,000 | 10,000 | 6,751 | -30 | -8 | 18 |  |
|  |  |  |  | 0,006 | 0,011 | 1486 | 0,003 | 0,000 | 0,000 | 9,800 | 6,680 | 32 | -4 | 18 |  |
|  |  |  |  | 0,000 | 0,000 | 10537 | 0,000 | 0,000 | 0,002 | 7,000 | 5,454 | 20 | -56 | -20 |  |
|  |  |  |  | 0,198 | 0,265 | 352 | 0,102 | 0,065 | 0,074 | 4,900 | 4,244 | 32 | 2 | -28 |  |
|  |  |  |  | 0,124 | 0,200 | 475 | 0,062 | 0,097 | 0,102 | 4,800 | 4,125 | 12 | -28 | 58 |  |
|  |  |  |  | 0,680 | 0,930 | 52 | 0,528 | 0,377 | 0,393 | 4,100 | 3,654 | 22 | 36 | 8 |  |
|  |  |  |  | 0,528 | 0,752 | 111 | 0,347 | 0,426 | 0,406 | 4,000 | 3,601 | -12 | -88 | 10 |  |
|  |  |  |  | 0,834 | 0,930 | 8 | 0,833 | 0,678 | 0,663 | 3,700 | 3,346 | -68 | -18 | 6 |  |
|  |  |  |  | 0,809 | 0,930 | 14 | 0,766 | 0,682 | 0,663 | 3,700 | 3,342 | -52 | -62 | -30 |  |
|  |  |  |  | 0,860 | 0,930 | 3 | 0,909 | 0,702 | 0,673 | 3,600 | 3,321 | -18 | -6 | -34 |  |
|  |  |  |  | 0,805 | 0,930 | 15 | 0,757 | 0,763 | 0,741 | 3,600 | 3,254 | -36 | -2 | -24 |  |
|  |  |  |  | 0,854 | 0,930 | 4 | 0,891 | 0,825 | 0,865 | 3,500 | 3,178 | -20 | 38 | 4 |  |
|  |  |  |  | 0,866 | 0,930 | 2 | 0,930 | 0,857 | 0,919 | 3,400 | 3,134 | -20 | -4 | -36 |  |
| **Case 5** | Grey matter reduction | 0,000 | 14 | 0,000 | 0,000 | 19894 | 0,000 | 0,000 | 0,001 | 8,041 | 5,943 | -53 | -69 | -9 |  |
|  |  |  |  | 0,001 | 0,003 | 4300 | 0,000 | 0,040 | 0,078 | 5,202 | 4,413 | 11 | -1 | 65 |  |
|  | Hypometabolism | 0,475 | 2 | 0,000 | 0,000 | 37018 | 0,000 | 0,000 | 0,000 | 15,400 | 65535,000 | -54 | -58 | 20 |  |
|  |  |  |  | 0,555 | 0,507 | 65 | 0,507 | 0,109 | 0,072 | 4,600 | 3,993 | 52 | -70 | 14 |  |
|  | Preserved metabolism | 0,798 | 1 | 0,000 | 0,000 | 68590 | 0,000 | 0,000 | 0,000 | 17,300 | 65535,000 | 34 | 0 | -28 |  |
